# Stem cell sources for tooth regeneration: current status and future prospects

**DOI:** 10.3389/fphys.2014.00036

**Published:** 2014-02-04

**Authors:** Keishi Otsu, Mika Kumakami-Sakano, Naoki Fujiwara, Kazuko Kikuchi, Laetitia Keller, Hervé Lesot, Hidemitsu Harada

**Affiliations:** ^1^Division of Developmental Biology and Regenerative Medicine, Department of Anatomy, Iwate Medical UniversityYahaba, Japan; ^2^Division of Special Care Dentistry, Department of Developmental Oral Health Science, Iwate Medical UniversityMorioka, Japan; ^3^INSERM UMR 1109, team “Osteoarticular and Dental Regenerative NanoMedicine”, Faculté de Médecine, Université de StrasbourgStrasbourg, France; ^4^Faculté de Chirurgie dentaire, Université de StrasbourgStrasbourg, France

**Keywords:** stem cells, iPS cells, tooth regeneration, tissue engineering, bioengineered tooth, teratomas, neural crest

## Abstract

Stem cells are capable of renewing themselves through cell division and have the remarkable ability to differentiate into many different types of cells. They therefore have the potential to become a central tool in regenerative medicine. During the last decade, advances in tissue engineering and stem cell-based tooth regeneration have provided realistic and attractive means of replacing lost or damaged teeth. Investigation of embryonic and adult (tissue) stem cells as potential cell sources for tooth regeneration has led to many promising results. However, technical and ethical issues have hindered the availability of these cells for clinical application. The recent discovery of induced pluripotent stem (iPS) cells has provided the possibility to revolutionize the field of regenerative medicine (dentistry) by offering the option of autologous transplantation. In this article, we review the current progress in the field of stem cell-based tooth regeneration and discuss the possibility of using iPS cells for this purpose.

## Introduction

Teeth consist of multiple hard tissues, including enamel, dentin, and cementum, and have an integrated attachment complex with alveolar bone through the periodontal ligament. After completion of tooth formation, the only vascularized tissue containing nerves is the dental pulp, which is encased in mineralized dentin. Because teeth have multiple functions, including feeding, articulation, and esthetics, their loss can cause not only physical but also psychological suffering that compromises an individual's self-esteem and quality of life (Pihlstrom et al., 2005; Polzer et al., 2010; Jung et al., 2011).

With aging, the number of people who lose their teeth increases. Furthermore, the number of people who have more than five congenitally missing adult teeth has increased (Mayama et al., 2001). Thus, tooth loss is a major challenge in contemporary dentistry and accounts for a large part of daily dental practice. Currently, missing teeth are restored using dentures or dental implants prepared from synthetic materials. Although these prostheses serve the purpose, denture therapy is associated with complications such as denture-induced stomatitis and traumatic ulcers (Holm-Pedersen et al., 2008). The use of dental implants may also lead to surgery failure because of many factors that interfere with osseointegration (Esposito et al., 1998). To overcome these shortcomings, the novel approach of stem cell-based tooth regeneration has been suggested as an alternative, considering the advances in tissue engineering and stem cell biology.

Recent progress in tissue engineering techniques and stem cell research has provided important insights for improving tooth regeneration. The main concept in current tooth regeneration is to mimic the natural tooth development process either *in vitro* or *in vivo* using stem cells. Because tooth development is characterized by a sequential reciprocal epithelial–mesenchymal interaction between oral epithelial and neural crest (NC)-derived dental ectomesenchymal cells (Thesleff and Sharpe, 1997), numerous studies have attempted to find an optimal source of stem cells that have the potential to differentiate into these cells or their progeny. In particular, the recent discovery of induced pluripotent stem (iPS) cells, which have been genetically reprogrammed to an embryonic stem cell (ESC)-like state, has had a major impact in this field (Takahashi and Yamanaka, 2006). In this review, we focus on the important previous findings in the study of tooth regeneration using stem cells and discuss the potential of iPS cells for tooth regeneration in light of recent results obtained by our group.

## Current stem cell-based tooth regeneration

Stem cells are unspecialized cells defined as clonogenic cells that have the capacity for self-renewal and the potential to differentiate into one or more specialized cell types. (Weissman, 2000; Slack, 2008). Their microenvironment, composed of heterologous cell types, extracellular matrix, and soluble factors, enables them to maintain their stemness (Watt and Hogan, 2000; Spradling et al., 2001; Scadden, 2006). Because of their unique properties, stem cells have the potential to be important in tissue engineering strategies for the regeneration of diseased, damaged, and missing tissues and even organs. In general, stem cells can be divided into three main types: ESCs that are derived from embryos; adult stem cells that are derived from adult tissue; and iPS cells that are generated artificially by reprogramming adult somatic cells so that they behave like ESCs. In this section, we outline recent results obtained using ESCs and adult stem cells for tooth regeneration.

### ESCs

The isolation and expansion of murine ESCs in the 1980s ignited interest in regenerative medicine research (Evans and Kaufman, 1981). ESCs are pluripotent stem cells derived from the undifferentiated inner cell mass of the blastocyst (an early stage of embryonic development) and they continue to grow indefinitely in an undifferentiated diploid state when cultured in optimal conditions in the presence of a feeder layer and leukemia inhibitory factor (LIF). The study of ESCs has gained further interest with the successful establishment of primate and human ESCs (Thomson et al., 1995, 1998; Shamblott et al., 1998; Reubinoff et al., 2000), which can differentiate into derivatives of all three primary germ layers: ectoderm, endoderm, and mesoderm (Evans and Kaufman, 1981; Thomson et al., 1998). Because of the pluripotency of ESCs, several attempts have been made to use them to functionally regenerate cardiomyocytes, dopaminergic neurons, and pancreatic islets in animal models, keeping in view future clinical applications (Lumelsky et al., 2001; Kim et al., 2002; Laflamme et al., 2007; Van Laake et al., 2008). In dentistry, ESCs have been used for oral and craniofacial regeneration, including mucosa, alveolar bone, and periodontal tissue regeneration (Roh et al., 2008; Inanç et al., 2009; Ning et al., 2010; Shamis et al., 2011). Ohazama et al. (2004) demonstrated that after recombination with embryonic day (E)10 oral epithelium, ESCs expressed the unique set of genes for odontogenic mesenchymal cells, such as Lhx7, Msx1, and Pax9, suggesting that ESCs can respond to inductive signals from embryonic dental epithelium. Although these approaches have the potential to be useful for tooth regeneration and for understanding basic tooth development, it will be necessary to address several major issues before they can be implemented in clinical practice, including possible tumorigenesis (teratoma formation) when transplanted, ethical issues regarding the use of embryos, and allogeneic immune rejection.

### Adult stem cells in dental tissues

Adult stem cells have been identified in many tissues and organs and have been shown to undergo self-renewal, to differentiate for the maintenance of normal tissue, and to repair injured tissues. The first adult stem cells isolated from dental tissues were dental pulp stem cells (DPSCs) (Gronthos et al., 2000). These cells have a typical fibroblast shape and express markers similar to those of mesenchymal stem cells (MSCs). When transplanted with hydroxyapatite/tricalcium phosphate (HA/TCP) powder in immunocompromised mice, they formed a dentin-like structure lined with odontoblast-like cells that surrounded a pulp-like interstitial tissue (Gronthos et al., 2000). DPSCs could differentiate *in vitro* into other mesenchymal cell derivatives such as odontoblasts (D'Aquino et al., 2008), adipocytes, chondrocytes, and osteoblasts (D'Aquino et al., 2007; Koyama et al., 2009; Yu et al., 2010) and could also differentiate into functionally active neurons (Arthur et al., 2008, 2009). MSC-like cells have also been isolated from the dental pulp of human deciduous teeth [stem cells from human exfoliated deciduous teeth (SHEDs)] (Miura et al., 2003). They have the ability to differentiate *in vitro* to neuron-like cells, odontoblasts, osteoblasts, and adipocytes, show higher proliferation rates and increased numbers of population doublings compared with DPSCs, and can form spherical aggregations. When these cells are transplanted mixed with HA/TCP *in vivo*, they can form dentin and bone but not dentin–pulp complexes. Comparison of the gene expression profiles of DPSCs and SHEDs demonstrated that 4386 genes were differentially expressed by two-fold or more (Nakamura et al., 2009). In addition to genes that participate in pathways related to cell proliferation and extracellular matrix formation, FGF, transforming growth factor (TGF)-β, and collagen I and III showed a higher level of gene expression in SHEDs than in DPSCs. Cordeiro et al. (2008) suggested that SHEDs could be the ideal source of stem cells for repairing damaged teeth or for induction of bone formation.

Stem cells from the apical papilla (SCAPs) are found in the papilla tissue in the apical part of the roots of developing teeth. The third molars and teeth with open apices are an important source of SCAPs. These cells have the potential to differentiate into osteoblasts, odontoblasts, and adipocytes and show higher rates of proliferation *in vitro* compared with DPSCs (Sonoyama et al., 2006, 2008; Huang et al., 2008). Transplantation of SCAPs and periodontal ligament stem cells (PDLSCs) into tooth sockets of minipigs allowed the formation of dentin and periodontal ligament (Sonoyama et al., 2006). Dental follicle stem cells (DFSCs) have also been isolated from the follicles of developing third molars (Morsczeck et al., 2005). They can differentiate into osteoblasts, adipocytes, and nerve-like cells *in vitro* (Kémoun et al., 2007; Coura et al., 2008; Yao et al., 2008) and form cementum and periodontal ligament *in vivo* (Handa et al., 2002; Yokoi et al., 2007).

Future therapeutic approaches for the restoration of damaged dentin, pulp, cementum, and periodontal ligaments may make use of autologous stem cells such as DPSCs, SHEDs, SCAPs, and DFSCs that have been stored after removal from the patient.

Dental epithelial stem cells were identified in the continuously growing rodent incisor (Harada et al., 1999). These cells are maintained in the stem cell niche located at the apical end of the incisor, named the “apical bud” region, and they constantly produce enamel-secreting ameloblasts through interaction with mesenchymal cells (Harada et al., 1999). FGF10, Notch, and Sprouty have been suggested to play a role in the continuous growth of rodent incisors and the maintenance of dental epithelial stem cells (Harada et al., 1999; Tummers and Thesleff, 2003; Klein et al., 2006; Yokohama-Tamaki et al., 2006). Although dental epithelial stem cells appear to be attractive for the regeneration of enamel-forming ameloblasts in rodents, this stem cell niche may be specific to rodent incisors; these cells differ from all human teeth, in which dental epithelial stem cells and their progeny are lost after eruption of the tooth.

The epithelial rests of Malassez (ERMs) are quiescent epithelial remnants of Hertwig's root sheath (HERS) that remain in the adult tooth and play a role in cementum repair and regeneration (Rincon et al., 2006). A recent study demonstrated that ERMs contain a unique population of stem cells that are capable of undergoing epithelial–mesenchymal transition and differentiate into diverse lineages indicative of mesodermal and ectodermal origin, including bone, fat, and cartilage as well as neuron-like cells (Xiong et al., 2013). In addition, ERMs can be induced to form enamel-like tissues after transplantation into athymic rat omentum with primary dental pulp cells (Shinmura et al., 2008), suggesting that the stem cells in ERMs may be able to regenerate enamel.

### Adult stem cells in non-dental tissues

Although most adult stem cells in non-dental tissues have generally been considered to be limited to specific cell fates, recent studies have demonstrated that they have plasticity and can differentiate into cell types derived from different germ layers. In particular, bone marrow-derived adult stem cells have shown considerable capacity to differentiate into diverse cell types such as endothelium, neural tissue, liver, and heart (Asahara et al., 1997; Lagasse et al., 2000; Mezey et al., 2000; Orlic et al., 2001). Notably, MSCs derived from bone marrow can respond to inductive stimulation from dental epithelium and contribute to tooth regeneration (Ohazama et al., 2004). Recombination between odontogenic-inducing epithelium and bone marrow-derived cells has been demonstrated to involve the expression of odontogenic genes such as Pax9, Msx1, and Lhx7 and the formation of a tooth crown with organized enamel, dentin, and pulp surrounded by bone after transplantation under the mouse kidney capsule (Ohazama et al., 2004). Furthermore, c-kit-enriched bone marrow-derived cells were shown to be able to differentiate into ameloblast-like cells (Hu et al., 2006b).

The prospective stem cells described above have shown remarkable capability for tooth regeneration. However, with regard to clinical application, they share the common obstacles of ethical concern arising from their embryonic origin, the risk of tumorigenesis, and the possibility of immune rejection after allogeneic transplantation. The development of iPS cells may overcome many of these issues because of their properties, and iPS cell-derived odontogenic cells can be expected to play significant roles in future strategies for clinical translational research on tooth regeneration.

## iPS cells

Generating patient-specific pluripotent stem cells with properties similar to those of ESCs has long been a central aim in research on stem cell-based regenerative medicine. Through global changes in the epigenetic and transcriptional environment, nuclear reprogramming reverses cell fate, converting differentiated cells back to the undifferentiated state (Jaenisch and Young, 2008). In somatic cell nuclear transfer (SCNT), also referred to as therapeutic cloning, the nucleolus of a somatic cell is transferred to the cytoplasm of an enucleated egg to create a blastocyst genetically identical to the parental source and to derive pluripotent ESC-like stem cells (Hochedlinger and Jaenisch, 2006). However, SCNT still needs the donor oocyte to direct the reprogramming of the somatic cell, and most cloned animals exhibit severe phenotypic and gene expression abnormalities (Humpherys et al., 2002; Ogonuki et al., 2002; Tamashiro et al., 2002). Therefore, SCNT is not a feasible option for cell-based transplantation. Although the mechanism by which transformation occurs and the mediators of nuclear reprogramming are largely undefined, the search for factors that are able to induce complete nuclear reprogramming has provided recent breakthroughs in the development of successful iPS technologies.

In 2006, Takahashi and Yamanaka reported the successful derivation of iPS cells from embryonic and adult mouse fibroblasts through the ectopic co-expression of only four genes: Oct4, Sox2, Klf4, and c-Myc (Takahashi and Yamanaka, 2006). The expression of these genes was sufficient to reprogram somatic cells to an ESC-like pluripotent state. Tissues from different species such as mice (Takahashi and Yamanaka, 2006), rats (Liao et al., 2009), rhesus monkeys (Liu et al., 2008), and humans (Takahashi et al., 2007) have been used as source materials for iPS cell line generation. Successful reprogramming also quickly translated to a wide variety of other cell types, including pancreatic β-cells (Stadtfeld et al., 2008), neural stem cells (Eminli et al., 2008) mature B cells (Hanna et al., 2008), stomach and liver cells (Aoi et al., 2008), melanocytes (Utikal et al., 2009), adipose stem cells (Sun et al., 2009), and keratinocytes (Maherali and Hochedlinger, 2008), demonstrating a universal capacity to alter cellular identity. In dentistry, iPS cells have been generated from many types of dental tissues/cells, including SHEDs, SCAPs, DPSCs, tooth germ progenitor cells (TGPCs), buccal mucosa fibroblasts, gingival fibroblasts, and periodontal ligament fibroblasts (Egusa et al., 2010; Miyoshi et al., 2010; Oda et al., 2010; Tamaoki et al., 2010; Yan et al., 2010; Wada et al., 2011). DPSCs show much higher reprogramming efficiency than the conventionally used dermal fibroblasts and high expression of endogenous reprogramming factors such as c-Myc and KLF4 and/or ESC marker genes (Tamaoki et al., 2010). Because these cells are easily accessible by dentist, iPS cells generated from dental tissues are expected to be a promising cell source for tissue regeneration.

iPS cells have shown pluripotency similar to that of ESCs. They can produce cells from all three germ layers *in vitro* and form teratomas when injected into immunodeficient mice and can contribute to chimera formation (Takahashi and Yamanaka, 2006). Murine iPS cells also fulfill the strict pluripotency criteria for contribution to the germline (Okita et al., 2007) and tetraploid embryo complementation (Woltjen et al., 2009). Moreover, they can maintain self-renewal when cultured under conditions similar to those used for ESCs. Hence, iPS cells are often described as indistinguishable from ESCs. However, the question of whether iPS cells and ESCs are molecularly and functionally equivalent is raised by the artificial nature of induced pluripotency. Recent analyses have shown a high degree of similarity between ESCs and iPS cells in terms of global gene expression and histone methylation (Maherali et al., 2007; Okita et al., 2007; Wernig et al., 2007; Mikkelsen et al., 2008). However, substantial differences between them have also been reported. In addition, other studies have indicated that iPS cells retain an epigenetic memory of their former phenotype that can limit their differentiation potential (Kim et al., 2010; Polo et al., 2010). Therefore, further study of iPS cells and ESCs is required to determine whether differences between them may affect their differentiation potential and their overall safety and efficiency after transplantation.

There are several advantages of using iPS cells for regenerative medicine. Their use can overcome the ethical and political issues associated with the use of embryonic cells. They can be used as autologous and patient-specific cells, which eliminates issues related to the immune rejection of grafts, and can thus be expected to become the major tool in the advancement of personalized medicine (Ferreira and Mostajo-Radji, 2013). Furthermore, iPS cell production can easily be scaled up, which essentially provides an unlimited source of cells for clinical applications, in contrast with adult stem cells.

In addition to regenerative medicine, newly emerging applications of iPS cells are related to *in vitro* disease modeling and drug screening (Ebert et al., 2012). Tissue-specific iPS-derived cells generated from patients with complex genetic defects can be used to model diseases in studies to elucidate the complex mechanisms underlying various diseases and to search for new drugs. Primary human cells carrying the disease of interest are usually difficult or impossible to isolate, and even if it is possible to isolate them, in most cases, the cells do not proliferate adequately to produce sufficient numbers of cells for analysis. In contrast, iPS cells derived from the patient can proliferate abundantly and differentiate into cells that represent the pathological character of the disease. Numerous groups have reported the creation of iPS cells specific for various diseases, including Parkinson's disease, amyotrophic lateral sclerosis, and familial dysautonomia, in studies to elucidate the mechanism of their development and progression and to search for suitable drugs (Dimos et al., 2008; Park et al., 2008; Lee et al., 2009).

Another therapeutic potential of iPS cells has been demonstrated in proof-of-principle studies. Hanna et al. (2007) used a humanized mouse model of sickle cell anemia to determine the repair potential of progenitor cells derived from autologous iPS cells. Fibroblasts from the diseased mice were reprogrammed into an iPS clone and the mutant gene was corrected by homologous recombination; the pluripotent cells then differentiated into hematopoietic progenitors and were transplanted back into the mice. This therapy resulted in substantial improvement of symptoms. In another milestone study, healthy iPS-derived dopaminergic neurons were implanted into the brain of a rat model of Parkinson's disease. The implanted cells were functionally integrated and the disease condition was improved (Wernig et al., 2008).

## Differentiation of iPS cells into epithelial stem/progenitor cells during teratoma formation

Teratomas that occur naturally in the ovaries are a useful tool for studying the development of tissues and organs because they consist of a variety of tissue elements derived from two or more embryonic germ layers (Linder et al., 1975). They have been shown to contain ectodermal appendages, such as teeth and hair, and are a unique material for investigating the mechanisms involved in morphogenesis. Therefore, although tumorigenesis may be a critical issue in the clinical application of iPS cells, these teratomas should provide an excellent model for investigating tooth formation and organogenesis and lead to novel bioengineering approaches in regenerative medicine (Gerecht-Nir et al., 2004; Nussbaum et al., 2007). We therefore examined the processes of epithelial histogenesis and the properties of epithelial tissues and whether or not epithelial stem/progenitor cells, which have the capacity to induce tooth organogenesis, were found in iPS-derived teratomas (Kishigami et al., 2012). After mouse iPS cells were transplanted subcutaneously, iPS cell-derived teratomas (days 7, 14, and 21) were evaluated histologically. In terms of the histomorphological features of the epithelium, compact epithelial mass structures composed of non-polarized cells were dominant during early teratoma growth (day 7), whereas mature structures, such as pseudostratified ciliated epithelium and keratinized stratified squamous epithelium, increased as the teratomas developed (days 14–21) (Kishigami et al., 2012). Furthermore, other mature tissues, such as bone and cartilage, became evident in late teratomas (day 21) (Kishigami et al., 2012). These results suggest that the processes observed during epithelial histogenesis in iPS cell-derived teratomas may mimic those occurring in normal embryonic development and provide a useful model for studying the formation of tissue structures during early development.

To study the presence of epithelial stem/progenitor cells in iPS cell-derived teratomas, immunohistochemical analysis was performed using antibodies for the epithelial stem/progenitor cell markers p63 and CD49f and dental epithelial cell marker keratin 14 (K14) (Salmivirta et al., 1996; Pellegrini et al., 2001; Kawano et al., 2004; Laurikkala et al., 2006). K14 and p63 were detected in epithelial masses, basal layer of stratified epithelium, and pseudostratified epithelium. CD49f was detectable in all epithelium types from day 7; in particular, it was strongly expressed in epithelial masses and basal layer of stratified epithelium (Kishigami et al., 2012). These results provide important insights into the development of epithelial tissues during spontaneous differentiation of iPS cells *in vivo*.

However, regardless of the presence of putative epithelial stem/progenitor cells, iPS cell-derived teratomas that formed in these conditions did not contain teeth, and no tooth germ-like structures could be found (*n* = 10, unpublished data). This suggests that a specific signaling network for tooth organogenesis is missing.

## Approaches to tooth regeneration using iPS cells

Because of recent advances in tissue engineering technology, functional teeth can be formed from dissociated tooth germ cells. Several groups have demonstrated that it is possible to produce biological teeth similar in appearance to natural teeth on the basis of tissue–cell or cell–cell recombination using embryonic tooth germ cells (Hu et al., 2005, 2006a; Nakao et al., 2007; Honda et al., 2008; Nait Lechguer et al., 2008, 2011). In addition, using tissue/cell recombination techniques, non-dental stem cells such as ESCs, neural stem cells, and bone marrow-derived cells have been shown to respond to inductive signals from embryonic dental epithelium (Ohazama et al., 2004). Depending on the stage, dental epithelium or mesenchyme from the tooth germ has an inductive potential for differentiating even non-dental stem cells into odontogenic cells.

**Figure 1:**
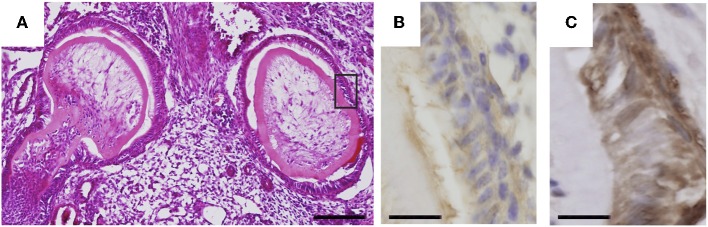
**Odontogenic response of undifferentiated iPS cells.** DsRed-expressing iPS cells were subcutaneously transplanted together with E14.5 mouse first molar mesenchyme cells. **(A)** H-E staining showing tooth like structures formed in iPS cell-derived teratoma. Bar = 100 μm. **(B)** Immunostaining for DsRed in the area shown by a rectangle in **(A)**. Bar = 10 μm. **(C)** Immunostaining for amelogenin in the serial section shown in **(B)**. Bar = 10 μm.

To investigate whether dental mesenchymal cells in the tooth germ could induce undifferentiated mouse iPS cells to form dental epithelial cells, DsRed-expressing iPS cells were combined with E14.5 dental mesenchyme and transplanted together with collagen sponges under the kidney capsule in immunodeficient mice. Four weeks after transplantation, tooth germ-like structures in iPS cell-derived teratomas were observed, and iPS cells expressed an ameloblast marker, amelogenin, indicating that iPS cells had differentiated into ameloblasts (Figure 1). However, the results of these transplantation experiments had poor reproducibility (<10%) and the numbers of tooth germ-like structures in the teratomas were very small (<2 per teratoma). Therefore, it appeared that more specific and suitable exogenous signals would be necessary to induce undifferentiated iPS cells to acquire odontogenic characteristics.

**Figure 2:**
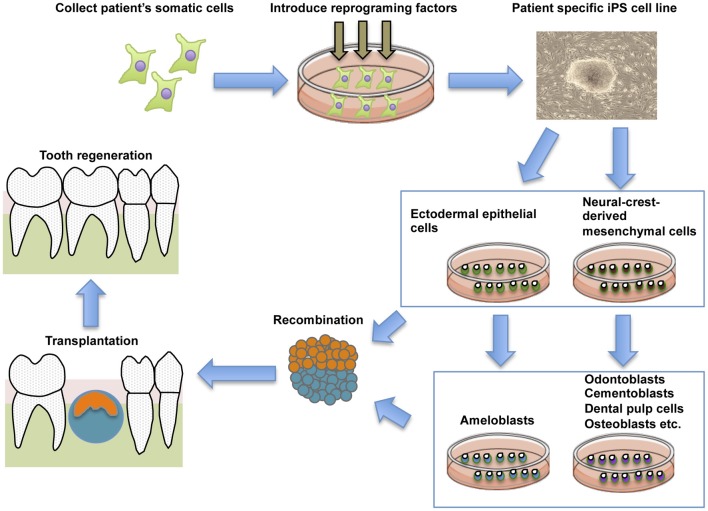
**General schematic representation of the current strategy for whole tooth regeneration using iPS cells.** The patient's somatic cells are harvested. Reprogramming conditions/factors are introduced to induce self-renewal and pluripotency, and patient-specific iPS cells are established. iPS cells are induced to form ectodermal epithelial cells and neural crest-derived mesenchymal cells, and they are further induced to form odontogenic cells *in vitro*. The two cell populations are combined by direct contact, mimicking the *in vivo* arrangement. Interaction of these cells leads to formation of an early-stage tooth germ. Once transplanted into the mouth, the recombinants develop and lead to functional recovery from tooth loss.

Tooth development is controlled by reciprocal interactions between dental mesenchymal cells derived from NC and dental epithelial cells derived from ectodermal epithelium (Thesleff and Sharpe, 1997; Jernvall and Thesleff, 2000). Epithelial–mesenchymal interactions also control the terminal differentiation of odontoblasts and ameloblasts (Ruch et al., 1995; Imai et al., 1996). Thus, as a new strategy for tooth regeneration, we speculated that ectodermal epithelial cells and NC cells induced from iPS cells could be the optimal cell source for the regeneration of whole teeth (Figure 2).

**Figure 3:**
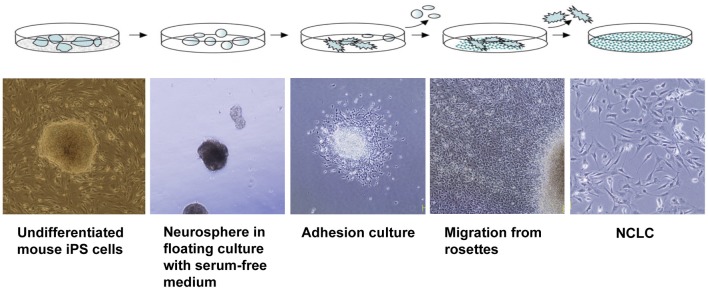
**Protocol for induction of iPS cells into NC cells.** iPS cells were differentiated in suspension to form neural spheres. The spheres spontaneously attached and formed rosette-like structures. The cells migrated away from the rosette. The migratory cells formed a uniform population of NCLCs. These images are reproduced with permission from a study reported in *Stem Cells and Development* (Otsu et al., 2012b).

A protocol for differentiation to NC (Figure 3), originally developed for human ES cells (Lee et al., 2007; Bajpai et al., 2009), efficiently induced mouse iPS cells to differentiate into neural crest-like cells (NCLCs) (Otsu et al., 2012b). These NCLCs expressed several NC cell markers, including AP-2α, Wnt-1, and p75^NTR^, and an MSC marker (Stro-1). Pax3, Snail, and Slug (NC-specific transcription factors), as well as human natural killer-1 (HNK-1, also known as CD57 and LEU7; a marker for migrating NC cells) showed higher expression in derived cells than in undifferentiated iPS cells (Otsu et al., 2012b).

Importantly, NCLCs did not form teratomas when they were injected subcutaneously together with collagen gel into immunodeficient mice, possibly because of the disappearance of Nanog, which is a marker of undifferentiated iPS cells (Okita et al., 2007) and subtly linked to tumorigenesis (Chiou et al., 2008). This result suggests that NCLCs derived from iPS cells can overcome the critical problem of tumorigenesis in the clinical application of iPS cell transplantation *in vivo* (Ben-David and Benvenisty, 2011).

**Figure 4:**
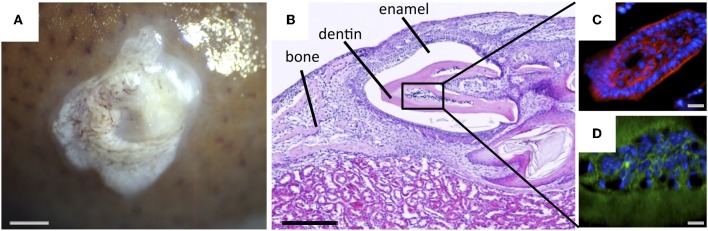
**Odontogenic response of NCLCs derived from iPS cells to dental epithelium. (A)** Image showing appearance of the recombinant between NCLCs and dental epithelium 2 weeks after transplantation under the kidney capsule. Bar = 500 μm. **(B)** Histological section of the recombinant showing tooth-like structure. Bar = 200 μm. Cells inside the dentin-like structure were positive for odontoblast markers DSP (**C**, Bar = 10 μm) and nestin (**D**, Bar = 25 μm).

When NCLCs were cultured in dental epithelial cell-conditioned medium, the expression of DSPP, a precursor protein of dentin sialoprotein (DSP), was significantly increased. Recombinant culture between NCLC and E14.5 dental epithelium in a collagen gel (Otsu et al., 2012a) or an agar-containing semi-solid medium (Hu et al., 2005; Keller et al., 2011) showed that NCLCs expressed the odontoblast marker DSP (Otsu et al., 2012b). Moreover, after transplantation under the kidney capsule in immunodeficient mice, the recombinant demonstrated calcified tooth germ-like structures with bone (Figure 4), indicating that iPS cell-derived NCLCs have the capacity to differentiate into odontoblasts via their reciprocal interaction with dental epithelium.

In addition to our recent results, several reports have demonstrated the potential of iPS cells for odontogenic differentiation. The hanging drop method on a collagen type-I scaffold combined with BMP-4 induced mouse iPS cells to form odontoblast-like cells without epithelial–mesenchymal interaction (Ozeki et al., 2013). These authors further demonstrated that integrin α2 in iPS cells mediated their differentiation into odontoblasts. BMP-4 was also shown to induce iPS cells to form both ameloblast-like and odontoblast-like cells when used with ameloblast serum-free conditioned medium (Liu et al., 2013). Moreover, co-culture with an ameloblastin-expressing dental epithelial cell line led to efficient induction of iPS cells into ameloblasts via neurotrophic factor NT-4 and BMP-4 signaling (Arakaki et al., 2012). These results strongly suggest that BMP-4 is a key molecule for odontogenic differentiation from iPS cells.

The ability of iPS cells to form tooth-like structures *in vivo* has also been confirmed by using recombination with tooth germ cells following transplantation under the kidney capsule (Wen et al., 2012; Cai et al., 2013). Furthermore, combination of iPS cells with enamel matrix derivatives was shown to greatly enhance periodontal tissue regeneration by promoting the formation of cementum, alveolar bone, and periodontal ligaments (Duan et al., 2011), indicating the possibility of iPS cell-based periodontal tissue regeneration.

## Concluding remarks

In this review, we discuss the potential of stem cell-based tooth regeneration, including the use of iPS cells. This field of research provides an attractive alternative to traditional and current practices for the replacement of missing teeth, such as implants and classic procedures based on synthetic materials. Because of rapidly increasing research efforts and progress, it is anticipated that clinically satisfactory functional tooth regeneration will be available in the near future. In particular, as part of a new technology, patient-specific iPS cells are a highly promising cell source for personalized regenerative dental medicine because of their potential to overcome the shortcomings of adult (tissue) stem cells and embryonic cells. The future establishment of this technique may considerably change therapeutic approaches to dental syndromes and diseases.

However, some challenges remain to be addressed before successful tooth regeneration can be achieved. For example, natural tooth development generally takes several years to complete in humans, which is too long to wait for a patient who needs regenerated teeth. Therefore, we should address this issue carefully when considering clinical applications. In addition, because morphology and size differ depending on the tooth type, these aspects need to be addressed. We also need to further develop efficient protocols to induce stem cells to form cell types *in vitro* that are relevant to the tissues and organs targeted for regeneration. To succeed in these challenges, further basic studies to elucidate the regulatory mechanisms of stem cells and tooth development are needed.

### Conflict of interest statement

The authors declare that the research was conducted in the absence of any commercial or financial relationships that could be construed as a potential conflict of interest.
